# Open Reduction and Internal Fixation of Extraarticular Scapular Neck and Body Fractures With Good Short Term Functional Outcome

**DOI:** 10.3389/fsurg.2019.00071

**Published:** 2019-12-17

**Authors:** Muhammad Azrin Mohd Asihin, Mohd Yazid Bajuri, Premganesh K. Ganaisan, Abdul Rauf Ahmad

**Affiliations:** ^1^Department of Orthopaedics, Hospital Tuanku Ja'afar, Seremban, Malaysia; ^2^Department of Orthopaedics and Traumatology, Universiti Kebangsaan Malaysia Medical Centre, Hospital Canselor Tuanku Mukhriz, Kuala Lumpur, Malaysia

**Keywords:** extraarticular, scapula fracture, decision making, functional outcome, open reduction

## Abstract

**Background:** The study aims to evaluate the short-term functional outcome of open reduction and internal fixation of extraarticular scapular neck and body fractures treated at our center over a period of 2-year duration at a tertiary referral center.

**Patients and Methods:** Between October 2015 and October 2017, we operated on 20 extraarticular scapular neck and body fracture. Ten were available for a one-off assessment. The mean time to surgery was 10 days (range, 3–19 days) and one-off assessment was done within 6–24 months (mean, 13 months). Indications includes (1) medial/lateral displacement (M/L) ≥ 20 mm, (2) M/L ≥ 15 mm if angular deformity ≥ 30°, (3) Angular deformity ≥ 45°, (4) Double lesion of superior shoulder suspensory complex (SSSC), with displacement ≥10 mm in both lesion, (5) Glenopolar angle (GPA) ≤ 22°, and (6) open scapular fracture. The functional outcome was measured using range motion and strength. Patient-reported outcome was assessed using Disabilities of the Arm, Shoulder and Hand (DASH) questionnaire and Short-Form-36 (SF-36) version 1.

**Results:** All patients achieved radiological union at time of assessment. None of the patients had post-operative complications. The mean DASH score was 19.3 (range, 1.7–39.3). All subcategories of SF-36 questionnaire scores between 70 and 89.6, with exception to REE (role limitations due to emotional problems) and REP (role limitations due to physical health). The average range of motion for the injured shoulders; forward flexion 157°, abduction 114°, and external rotation 42°. The strength of operated and non-operated shoulders, respectively, 6.5 and 8.1 kgF of forward flexion, 5.5 and 7.2 kgF of abduction, and 4.1 and 6.3 kgF of external rotation.

**Conclusion:** Open reduction and internal fixation of surgically indicated scapular neck and body fracture is feasible with predictably good functional outcome. The reduced external rotation ROM and strength may be due to the use of Classic Judet approach, however we do not enough data to support this.

## Introduction

Open reduction and internal fixation of extraarticular scapular fracture have not been given enough attention in comparison to intraarticular fracture until recent studies found that scapula is an integral part of the dynamic stabilizer of humerus and shoulder system (1, 2). In a systematic review of 520 patients with a scapular fracture from 22 case series report, 99% of isolated scapular body fracture and 83% of isolated scapular neck fracture were treated non-operatively, in comparison to 80% of intraarticular fracture being treated operatively (3).

Gosen et al. evaluated 22 patients with scapular body fractures treated conservatively (4). The injured shoulder range of motion was significantly reduced in all three directions, i.e., abduction, forward flexion and external rotation (*P* = 0.011, 0.004, 0.001) when compared to the contralateral uninjured shoulder. Also, he made a comparison between the multiple-injury (including intraabdominal injury) group with the single-injury (isolated scapular fracture) group and found that the injured shoulder range of motion worsened in the presence of multiple injuries. A similar unfavorable outcome was reported in another study involving 51 patients with extraarticular scapular fracture treated conservatively (5). The injured shoulder ROM (abduction, forward flexion, and external rotation) and strength in the same plane of motion were significantly reduced in comparison to the uninjured shoulder (*P* = 0.001).

Surgical treatment of an extraarticular scapular fracture is gaining popularity with the advancement of surgical technique, new implants, and objective measurement to assist in decision making and to measure outcome (6–11). Currently, available literature has shown good outcome with open reduction and internal fixation of an extraarticular scapular fracture, where it is indicated (10, 12, 13).

The goal of this study is to evaluate the short-term functional outcome of open reduction and internal fixation of extraarticular scapular neck and body fractures treated at a tertiary referral center.

## Patients and Methods

We included all extraarticular scapular fracture presented or referred to our center over a period of 2 year (October 2015 to October 2017) treated with open reduction and internal fixation. Patient with one or more surgical indications as stated by Anavian et al. were included. (11) Indications includes (1) medial/lateral displacement (M/L) ≥ 20 mm, (2) M/L ≥ 15 mm if angular deformity ≥30°, (3) Angular deformity ≥ 45°, (4) Double lesion of superior shoulder suspensory complex (SSSC), with displacement ≥10 mm in both lesion, (5) glenopolar angle (GPA) ≤ 22°, and (6) open fracture. Exclusions criteria includes scapular fracture with glenoid (articular surface) involvement or isolated acromion and coracoid fracture, patient with traumatic brain injury with poor recovery, an ipsilateral brachial plexus injury, and a contralateral upper limb fracture (Table 1).

**Table 1:** Inclusion and exclusion criteria.

**Inclusion criteria**	**Exclusion criteria**
1. Medial/lateral displacement (M/L) ≥ 20 mm 2. M/L displacement ≥ 15 mm if angular deformity is ≥30° 3. Angular deformity ≥ 45 4. Double lesion of superior shoulder suspensory complex (SSSC), with displacement ≥ 10 mm in both lesions. 5. Glenopolar angle (GPA) ≤ 220 6. Open scapular fracture	1. Glenoid fracture (articular involvement) 2. Isolated acromion/coracoid process fracture 3. Traumatic brain injury with poor recovery 4. Ipsilateral brachial plexus injury 5. Contralateral upper limb fracture

Written informed consent was obtained from each participant for the publication of this article. The retrospective study has been approved by The Hospital Board from Hospital Tuanku Ja'afar, Seremban, Negeri Sembilan, Malaysia for ethical purposes.

**Figure 1:**
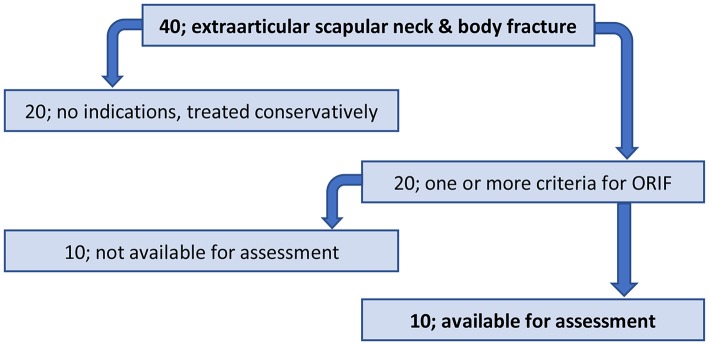
Patient selection process for one-off assessment.

We received a total of 40 patients with extraarticular scapular neck and body fracture. Half were treated conservatively and the other 20 fulfill one or more criteria for open reduction and fracture fixation. Of the 20 patients treated surgically, only 10 patients were available for the one-off assessment. The other 10 patients were either lost to follow up or refused to come for assessment. Final statistical analysis was made based on the 10 available patients. Mean time to the one-off assessment was 13 months (ranges 6–24 months) (Figure 1).

There were seven males and three females with a mean age of 43 years age (ranges 23–65 years old), and three patients aged above 50 years old (50, 59, and 65 years old). All fractures were the results of a high impact motor vehicle accident—seven extraarticular body of scapular fracture, and three extraarticular neck of scapular fracture. Five patients had an ipsilateral clavicle fracture, two had one or more ipsilateral rib(s) fracture treated conservatively, and the other two had a splenic injury treated conservatively. All ipsilateral clavicle fracture was plated in the same setting with scapular fracture fixation. None of the 10 patients had any injury to the contralateral upper limb (Table 2).

**Table 2:** Patients age, gender, time to surgery, and scapular injury type.

**Patient**	**Age, gender**	**Time to surgery (days)**	**Type**
1	65, Male	9	Body
2	37, Male	12	Neck
3	39, Male	10	Body
4	48, Female	7	Body
5	50, Male	5	Neck
6	35, Male	18	Body
7	45, Male	19	Neck
8	59, Male	3	Body
9	24, Female	7	Body
10	23, Female	7	Body

### Radiological Assessment

Standard plain radiograph for all patients includes scapular anteroposterior (Grashey view) and lateral (Scapular Y view). CT scan with a 3D reconstruction of the fractured scapular was requested when a displaced scapular fracture detected on the plain radiograph. Displacement and angulation of the fractured scapular measured and recorded by the treating surgeon based on the CT-3D reconstruction as this is a more accurate method (11). Patient with one or more indication was subjected to open reduction and internal fixation.

### Indications for Surgery

Anavian et al. validated a reliable technique to measure angulation and displacement of an extraarticular scapular fracture and confirm that plain radiograph is less reliable than CT scan in measuring the parameters (11). Coles et al. used these measurement techniques as a foundation to describes surgical indications of an extraarticular scapular neck and body fracture (10). He advocated surgical intervention if the fracture met one or more of these six indications; (1) medial/lateral displacement (M/L) ≥ 20 mm, (2) M/L ≥ 15 mm if angular deformity ≥ 300, (3) Angular deformity ≥ 45, (4) Double lesion of superior shoulder suspensory complex (SSSC), with displacement ≥10 mm in both lesion, (5) glenopolar angle (GPA) ≤ 220, and (6) open fracture. These parameters have gain popularity and used objectively to guide decision making in treating an extraarticular scapular fracture, and used as guidelines in studies involving similar fracture pattern (12–14). We used these six parameters to guide the treatment of all extraarticular scapular neck and body fracture at our center.

### Implants

**Figure 2:**
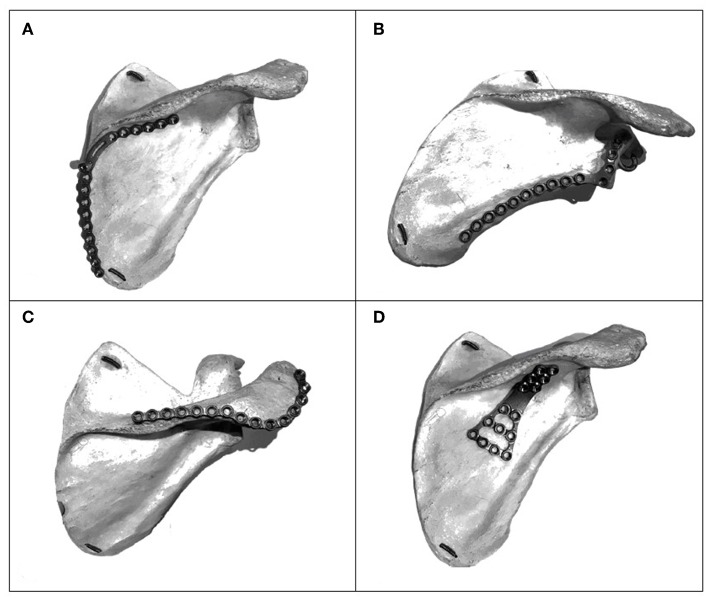
**(A)** Medial border anatomical locking plate. **(B)** Lateral border and neck of scapula plate. **(C)** Acromion and scapular spine plate. **(D)** Buttress plate for “floating” fragment.

In the past, we used a combination of implants such as 2.7 mm plates, 3.5 mm reconstruction plate, and 1/3 tubular plate. In recent years, we have designed our own 2 mm thick low-profile anatomical locking plate system for fixation of the extraarticular scapular fracture, using 2.5 mm cortical non-locking and 2.7 mm locking screw with the shortest length of 6 mm. This 6 mm short locking screw often used when plating the lateral and medial border of the scapular. The anatomical plates address the fractures at the medial border (Figure 2A), lateral border and surgical neck (Figure 2B), acromion and scapular spine (Figure 2C), a buttress plate for floating segment (Figure 2D), and a straight plate to be used where necessary. We often encountered fracture within the middle part of the scapular body which floats freely and can be frustrating to fix. We address this “floating” fragment using our own designed buttress plate. All the plates are bendable and can be cut to suit different fracture pattern.

### Surgical Approach

Patient with isolated scapular neck or body fracture was positioned in a semi-prone position, 45° lateral decubitus, with the fractured scapular facing upwards, and a small bolster placed in front to support the patient chest from falling flat on the operating table with the fractured scapula facing upwards, and a small bolster placed in front supporting the patient chest. The ipsilateral arm draped free into the surgical field to allow the arm used as a lever to mobilize the scapular when manipulation required to reduce the fracture. Patient with an ipsilateral clavicle fracture, however, was positioned in a floppy lateral. In this position, the patient can freely flip on either side to allow fixation of the clavicle before fixation of the scapular fracture. We perform clavicle fracture fixation before the scapula.

We utilized a Modified Judet approach for scapular fracture reduction and only extended to classic Judet when the fracture reduction is difficult and require further manipulation to reduce fracture displacement or in the presence of “floating” segment within the middle portion of the scapular body. We believe it is essential to restore the anatomy of the scapular blade to prevent scapulothoracic joint disorder such as snapping scapular syndrome resulting from the abnormal articulation of the scapulothoracic joint (15). The Modified Judet approach allows similar access to essential landmarks for fracture reduction and fixation with lesser soft tissue stripping, particularly the infraspinatus muscle and its blood supply (9, 16). A drain was left under the infraspinatus muscle before closing the skin.

### Post-operative Care

**Figure 3:**
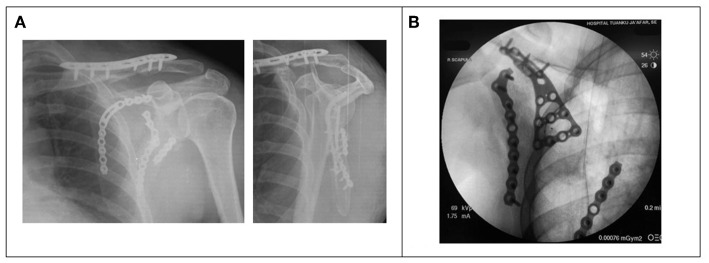
**(A)** Post-operative radiograph (AP and Y-view) showing clavicle plate, with medial and lateral locking plate *in situ*. **(B)** Fluoroscopy image of the Buttress plate used to press down “floating” segment of scapular fracture.

The drain discarded once the output is <10 ml per shift, which usually occur on day 2 following surgery. All patients were placed in an arm sling temporarily for comfort in the first 2 weeks. Passive range of motion exercise started once the drain removed, and active range of motion exercise begins in the third week post-operatively, with increasing resistance weekly after that. Follow up is done at 6 weeks, 3 months, 6 months, and 1-year mark. All patient reviewed with plain radiograph at every review (Figures 3A,B).

### Assessment of Outcomes

Patients were called in for a one-off evaluation at the Orthopedic clinic. Interview, physical examination, and test were all done by a single surgeon. Both injured and uninjured shoulder's range of motion (ROM) and strength were assessed using a reliable technique (8, 12). Abduction, forward flexion, and external rotation of the shoulder were assessed using a goniometer to determine the range of motion. A Jamar Hydraulic Hand-Held Dynamometer was used to assess the shoulder strength in the same direction of the assessed ROM (Table 3). The ROM of the injured shoulder was compared to the uninjured shoulder and recorded as a percentage of the uninjured shoulder (ROM or strength) (Table 4). Patient-reported outcomes was evaluated with Short Form-36 (version 1) and Disabilities of Arm Shoulder and Hand (DASH) questionnaire.

**Table 3:** Dynamometer strength measurements (kilogram-force; kgF).

**Strength (KgF)**	**Mean** **±** **Std Dev**		
	**Injured**	**Uninjured**	**Inj/Uninj (%)**
Forward flexion	6.5 + 2.0	8.1 + 2.8	80
Abduction	5.5 + 1.4	7.2 + 2.5	76
External rotation	4.1 + 1.2	6.3 + 2.0	65

**Table 4:** Range of motion measurement using goniometry.

**Range of motion (degree)**	**Mean** **±** **Std Dev**		
	**Injured**	**Uninjured**	**Inj/Uninj (%)**
Forward flexion	157 ± 13.6	174 ± 8.2	90
Abduction	114 ± 11.8	142 ± 5.3	80
External rotation	42 ± 7.2	60 ± 2.8	70

## Results

Average waiting time to surgery was 10 days (range 3–19). All patient achieves radiological union during the one-off assessment (6–24 months after surgery). All patients returned to work except for the two patients with a DASH score of 35 and 39.5, both aged 59 and 65, respectively. None of the patients developed post-operative complications such as infection, non-union and failure of internal fixation at time of assessment.

The patient-reported outcome was measured using the DASH score and the SF-36 questionnaire. The mean DASH score in our group of 10 patients, is 19.3 (ranging between 1.7 and 39.3). The SF-36 (version 1) scores for all parameters varies between 70 and 89.6 (healthy population ranges between 61 and 84) (6, 17), with exception to REE (role limitations due to emotional problems) and REP (role limitations due to physical health), scoring 26.7 and 20, respectively.

The ROM and strength of the injured shoulder were measured and recorded by the same operator. These values were compared to the uninjured shoulder. The functional outcome of surgery in the injured upper limb stated as a percentage of the uninjured upper limb ROM or strength. The average range of motion of the operated shoulder produced 90% (forward flexion), 80% (abduction), and 70% (external rotation) of the same average range of motion of the uninjured contralateral shoulder. The average strength test yield 80% (forward flexion), 76% (abduction), and 65% (external rotation) of the average strength of the uninjured shoulder.

The small sample size made it not possible for a statistical analysis to be done in our group of patients, hence we were unable to comment on the significance of our observed differences.

## Discussion

There was not much work done in the past comparing the outcome of operative and non-operatively treated extrascapular neck and body fracture. The surgical treatment became more popular recently with the advent of new and reliable techniques (7–13, 16, 18). These new techniques provide objective guidance to what is unacceptable displacement and angulation for any extraarticular scapular fracture, and therefore is indicated for surgical fixation. Good functional outcomes and return to function with operative treatment were reported in recent literature (12, 13, 19).

Herrera et al. reported good outcomes in 22 patients despite delay in operative treatment (range, 21–57 days). The abduction, forward flexion, and external rotation in the injured limb were found to be 94, 97, and 86% of the uninjured shoulder, respectively. The injured shoulder strength with hand-held dynamometer recorded average abduction of 83%, forward flexion of 73%, and external rotation of 73% in comparison to the uninjured contralateral shoulder. None of the 22 patients developed post-operative complication such as infection, non-union or failure of internal fixation. In the same institution, Schroder et al. studied 61 patients treated within 20 days following trauma and was able to reproduce similar findings. He reported that the injured shoulder average ROM ranges between 96 and 99% of the uninjured shoulder, while the shoulder strength score between 85 and 88% of the uninjured shoulder.

In our study, the mean range of motion (ROM) in the injured shoulder were 114° of abduction, 157° of forward flexion, and 42° of external rotation. These values were comparable to what were reported by Herrera et al. and Schroder et al. (abduction 106°, 106°; forward flexion 152°, 154°; external rotation 61°, 66°). The percentage of ROM in the injured shoulder compared to the non-injured shoulder; 90% of forward flexion, 80% of abduction, and 65% of external rotation. We noticed that there is reduced external rotation in our group of 10 patients. The possible contributing factor to this is the tendency to convert the surgical approach to Classic Judet approach in difficult scapular fracture reduction and cases with central floating fragment (four patient) requiring extended exposure of surgical fixation using the buttress plate (Figure 2D). However, a larger sample size in a future study is necessary to compare the functional outcomes between different surgical technique and different type of implant.

Schofer et al. reported a direct correlation between the reduced range of motion and the strength of the injured shoulder (5). He found that shoulder strength worsened as the limitation of motion in the respective plane increases. Similarly, we noticed the same finding in our patients, where the external rotation strength of injured shoulder is only 65% of the uninjured shoulder, in response to the reduced external rotation ROM i.e., 70% of the uninjured shoulder.

We reported mean DASH score of 19.3 (range 1.7–39.3) in our 10 patients. A DASH score between 0 and 29 is considered to be the point where the patient no longer considers their upper limb disorder is a problem (20). Past studies reported mean DASH score between 8.1 and 14 for all patients who underwent surgical interventions for scapular neck and body fractures (10, 13, 16, 18, 19, 21). The patient-reported functional outcome using SF-36 questionnaire scored between 70 and 89.6 (comparable to normal healthy population 61 to 84), with exception to two subcategories, i.e., REE (role limitations due to emotional problems) and REP (role limitations due to physical health). Questions number 13–19 of SF-36 representing both REE and REP has only two response which can either give a reported value of zero or 100 marks. The two subcategory were found to be less sensitive to the Malaysian population (22). The nature of the subcategory itself was thought to be relatively coarse role disability (17). Gosens et al. noted that patient with multiple fracture tend to have lower score in each subcategory of SF-36 questionnaire when compared to patients with single injury (scapular fracture only). Three of our patients sustained ipsilateral clavicle fracture, two had ipsilateral clavicle and ribs fracture, and another two had splenic injury treated conservatively.

Surgical fixation of scapular fracture allows early mobility and prevent complications such as restricted range of motion, reduced muscle power, pain, and residual scapular deformity (4, 23). In our experiences, fixation of the lateral and medial border of scapular body require a low profile plate with short screw length between 6 and 10 mm. In the past there were no specific implant for scapular fractures. We used a combination of 2.7 mm mini plates, 3.5 mm reconstruction plate, and 1/3 tubular plate. All of these implants are large, crude, and does not have suitable screw length to accommodate the thickness of the lateral and medial border of the scapular body. Our team designed a low profile 2 mm thick anatomical locking plate system for fixation of the extraarticular scapular fracture, using 2.5 mm cortical non-locking and 2.7 mm locking screw with the shortest length of 6 mm. This implant will be able to provide the needed mechanical stability, reduces the risk of implant failure, and loss of fracture reduction.

The scapular fracture tends to produce a poor functional outcome when treated conservatively, especially in the presence of multiple injuries such as intraabdominal injury and ipsilateral clavicle fracture (4), and this may be because an additional injury is likely to hinder early mobilization and physical therapy of the affected shoulder. We feel that patient with multiple fractures should be taken as a relative indication for surgical fixation of a scapular fracture.

Our study has limitations. The sample size is small and consist of only patients treated operatively and comparison was made with the contralateral uninjured shoulder within the same patients. Patients who were treated conservatively were not included. We therefore were unable to compare the functional outcomes between patients treated operatively and conservatively. The surgical approach was mixed (classic and modified Judet) and we do not have enough data to compare between the two surgical approach. In addition, the functional outcomes measurement was done by the treating surgeon and this may contribute to bias. However, an objective measurement of the ROM and strength were used to minimize biasness. A standard DASH score and SF-36 score questionnaire was used in all patients.

## Conclusion

With exception to the external rotation ROM and strength, we were able to demonstrate fairly good functional outcome in open reduction and internal fixation of displaced extraarticular scapular fracture. A future study with a larger sample size within a longer duration and the inclusion of patients treated conservatively in the data analysis will add more value to our findings.

## Ethics Statement

The studies involving human participants were reviewed and approved by the Hospital Board from Hospital Tuanku Ja'afar. The patients/participants provided their written informed consent to participate in this study.

## Author Contributions

MB, MM, PG, and AA: writing manuscript, collecting data, analyse data, and proof read.

### Conflict of Interest

The authors declare that the research was conducted in the absence of any commercial or financial relationships that could be construed as a potential conflict of interest.
